# Rice Straws With Different Cell Wall Components Differ on Abilities of Saccharification

**DOI:** 10.3389/fbioe.2020.624314

**Published:** 2021-01-20

**Authors:** Chen Chen, Xiaoxiao Deng, Weilong Kong, Mirza Faisal Qaseem, Shuai Zhao, Yangsheng Li, Ai-Min Wu

**Affiliations:** ^1^State Key Laboratory for Conservation and Utilization of Subtropical Agro-Bioresources, South China Agricultural University, Guangzhou, China; ^2^Guangdong Laboratory of Lingnan Modern Agriculture, South China Agricultural University, Guangzhou, China; ^3^Guangdong Key Laboratory for Innovative Development and Utilization of Forest Plant Germplasm, College of Forestry and Landscape Architectures, South China Agricultural University, Guangzhou, China; ^4^State Key Laboratory for Hybrid Rice, College of Life Sciences, Wuhan University, Wuhan, China; ^5^State Key Laboratory for Conservation and Utilization of Subtropical Agro-Bioresources, Guangxi Research Center for Microbial and Enzyme Engineering Technology, College of Life Science and Technology, Guangxi University, Nanning, China

**Keywords:** cell wall, glucose yield, pretreatment, *indica*, *japonica*

## Abstract

Rice straw has an enormous amount of biomass for energy use, but the complexity of the cell wall component hinders technical processes. Although belonging to rice straws, the straws from different varieties should be with different treatment strategies to obtain best energy efficiency. To confirm this hypothesis, 7 different rice varieties (RPY GENG, RIL269, RIL272, RIL31, RIL57, RIL06, LUOHUI 9) with different cell wall traits from RIL population were evaluated for their response toward different pretreatments. For *japonica* RPY GENG, 2% of H_2_SO_4_ acid was best pre-treatment while high acid (5% of H_2_SO_4_) pretreatment caused undue loss. For *Indica* LUOHUI 9 rice, high acid pretreatment was suitable, while RIL57 had maximum of glucose yield with high alkali (10% NaOH) pretreatment. High-concentration alkali pretreatment is the most convenient and effective pretreatment method for the treatment of unknown varieties of rice straws, because the lignin has been removed and has the lowest negative effects on the glucose yield under the high alkali condition. As the RILs used in this study vary considerably in their wall structure, an understanding of their response to different pre-treatments confirms our hypothesis and help us to understand the influence of different wall compositions on the final output.

## Introduction

Rice straw makes a significant contribution to lignocellulosic biomass and has great utilization value for bioethanol production in China. Many chemical and biological treatments are required for efficient conversion of rice straw to fermentable sugars which later can be converted to ethanol or other fuels (Takano and Hoshino, 2018). Rice straw has complex and heterogenous structure in which cellulose fibers are clumped with lignin and hemicellulose and thus a complex set of physical, chemical and biological pretreatments are required to break this complex structure (Binod et al., 2010). Although lot of work has been done on crop improvement for high biomass yield, improving the pretreatment process and using biotechnological methods to increase the saccharification capacity could also improve straw utilization (Tian et al., 2018). Existing studies have shown that many crops have unique biomass composition, but knowledge about difference in biomass composition between high-cultivated varieties derived from same parents is either lacking or only superficial (Chen et al., 2020; Samavi and Rakshit, 2020). For example, rice varieties cultivated in different regions of the world might have different straw cell wall composition, so application of one pretreatment technology for straw conversion to bioethanol is undesirable (Gao et al., 2005). Due to large-scale planting of different varieties, there is a need to explore appropriate pretreatment strategy basing on cell wall structures of rice straw in order to improve efficiency for obtaining maximum industrial benefits and environmental protection.

In previous studies, changes in pretreatment methods significantly affected the amount of reducing sugars. The results of wheat straw showed that the total reducing sugars under oxalic acid dihydrate treatment eventually reached 42%, however the glucose rate by dilute sulfuric acid treatment reached 90%. Not only that, the effects of multiple varieties under the same pretreatment show quite big difference as well (Tian et al., 2018). So many factors of the cell wall structures might affect pretreatment effect (Li et al., 2010; Zhang et al., 2011). For example, crystalline cellulose is not easily decomposed, and lignin and hemicellulose affect the accessibility of enzymes (Lenting and Warmoeskerken, 2001; Van Wyk, 2001; Qian et al., 2005; Brandt et al., 2013; Chen et al., 2020). Various pretreatment methods are available, including chemical biological and physical methods. For examples, ball milling increase the contact area of enzyme activity and are environmentally friendly, and alkaline treatment can fully destroy the dense structure of the cell wall, and acid treatment is also a more commonly used technique (Mcintosh and Vancov, 2010; Tian et al., 2018; Kellock et al., 2019). Previous studies have proposed the factors of cell wall composition influencing on sugar production, including cell wall porosity, cellulose crystallinity and lignin, and X/A. In fact, previous studies have shown that samples with genetically modified cell walls can improve the ability of biomass saccharification and ethanol production (Li et al., 2018; Alam et al., 2019). Many of the components in the cell wall are tightly connected. The purpose of pretreatment is therefore to physically or chemically destroy the stability of the straw cell wall and improve the efficiency of utilization. However, the best pretreatment methods for different varieties of *indica*-*japonica* hybrid rice are still worthy of further discussion.

Existing cultivated rice mainly belongs to two major groups i.e., *Indica* and *Japonica*. *Indica* rice is distributed in tropical and subtropical regions, and *japonica* rice is mainly distributed in temperate highlands (Gao et al., 2005). The cell wall composition of various rice straws differed significantly. Most comparison of preprocessing studies used cell wall gene related mutants, but there are few studies on cultivated rice. The cell wall structure of the recombinant inbred line populations of the hybrid offspring of *indica* rice and *japonica* rice showed great segregation in traits. In fact, the cell wall characteristics of straw are important for the pretreatment and products.

In this study, seven representative members out of big population of 272 recombinant inbred lines (RILs) developed after crossing *Indica* and *japonica* rice parents were randomly selected due to distinct cell wall components with gradient lignin composition. We used two pretreatment methods and six solution concentrations to destroy cell walls, and compared the enzymatic glucose yields of different samples under various pretreatment conditions. The purpose was to evaluate the enzymatic hydrolysis efficiency basing on different cell wall composition in the pretreatment process, to provide a more economic optimization idea for the utilization of different straws and to reduce utilization costs.

## Results

### Progeny Rice Populations Have Obvious Differences in Cell Wall Traits

**Figure 1:**
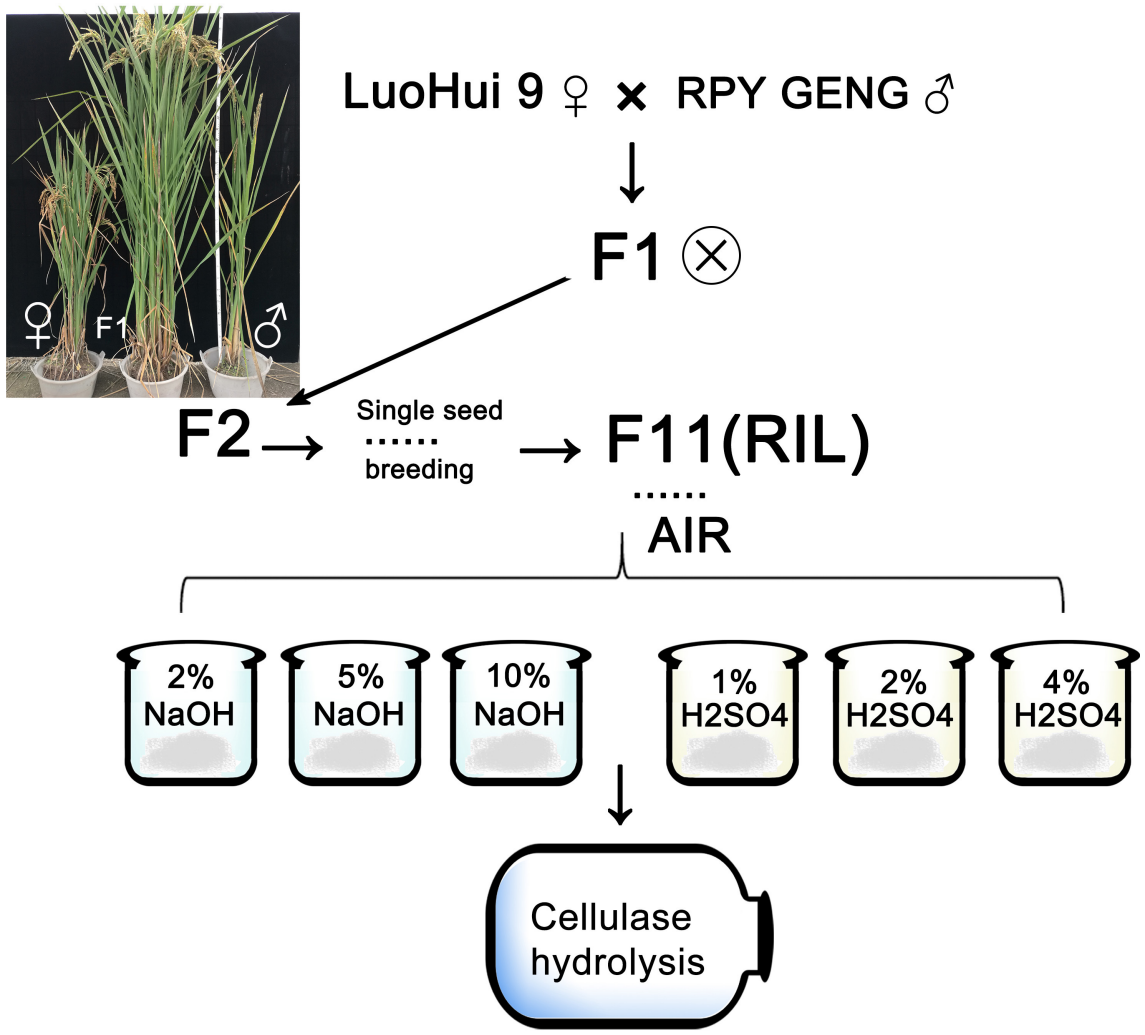
The source and process of recombined inbred populations of *indica* and *japonica* hybrids. The picture shows the parental (both sides) and some of the hybrid offspring. The mark next to F1 indicates self-crossing. The straw of F11 progeny RIL is used to prepare AIR, and the steps refer to “Methods.”

Basing on the theory that different cell wall composition of straw among rice varieties might explore suitable pretreatment strategies for best bioenergy products. Thus, the rice populations of different rice varieties were screened to find the straw samples with relatively big different cell wall composition. So, the rice varieties *indica* and *japonica* hybrids were screened. The offspring of the hybrids were self-crossed and single-grained. After several populations, 272 recombinant stable inbred lines were obtained (Figure 1). Initial screening of RIL population revealed that rice lines differ in their appearances, i.e., height, thickness. The rice straw grew normally in the field. Next, we measured the composition distribution of the straw cell wall of the entire population. The cellulose, hemicellulose and lignin contents in straw ranged from 30 to 50%, 10 to 20%, and 10 to 30% of whole population, respectively (Supplementary Figures 1A–C). Among all three components, the change in lignin content was relatively higher. This indicates that the lignin in rice varieties might be an important parameter for our hypothesis.

The change of straw component structure will inevitably affect the degree of decomposition. Generally, the cellulose and xylan can be degraded to glucose (Glu) and xylose (Xyl) in the cell wall after saccharification. The Glu/Xyl ratio measured for the entire population fluctuated around 3.0, indicating that the cellulose content in rice straw was higher. Among whole population, the RIL138 had highest value (7.42) of Glu/Xyl ratio while RIL244 had least value (1.86) (Supplementary Figure 1D). As it is known, xylose will be difficult to utilize for biofuel compared to glucose, so quilt large difference of Glu/Xyl ratio indicates that we should classify rice straws for high biofuel efficiencies.

### Selection of RILs Suitable for Studying the Effect of Lignin on Saccharification

The compactness and stability of the lignin structure make it one of the greatest obstacles to the use of straw cell walls. From the performance of 270 RILs, the correlation between different traits was not obvious (Supplementary Figure 2). Therefore, we randomly selected five samples with significant differences in lignin content as representative. The seven selected samples were first tested for cell wall structure by NREL standard. The results showed obvious difference in cell wall composition among the seven samples. The cellulose content of LUOHUI 9 (*indica* rice, female parent) was much lower than average of the offspring, only 27.99%, while the lignin content was higher. RPY GENG (*Japonica* rice, male parent) showed higher cellulose content and lower lignin content (Table 1). The lignin content of the both parents LUOHUI 9 and RPY GENG was at the maximum value of 28.06% and the minimum value of 14.92%, respectively (Table 1).

**Table 1:** The cell wall composition of the seven selected straw samples.

**Sample**	**Lignin%**	**Cellulose%**	**Hemicellulose%**	**Xyl%**	**Ara%**	**Gal%**	**X/A**
RPY GENG	14.92 ± 1.52	38.59 ± 3.44	18.74 ± 1.69	12.96 ± 1.43	4.15 ± 0.32	1.63 ± 0.22	3.13
RIL269	18.31 ± 0.91	41.64 ± 3.84	15.35 ± 0.84	8.57 ± 1.69	5.53 ± 0.49	1.25 ± 0.14	1.55
RIL272	20.29 ± 1.05	30.96 ± 5.57	17.64 ± 1.32	12.20 ± 1.01	4.67 ± 0.86	0.73 ± 0.13	2.62
RIL31	22.53 ± 2.33	31.40 ± 4.83	17.66 ± 0.98	10.02 ± 0.77	6.64 ± 0.91	1.00 ± 0.09	1.51
RIL57	22.08 ± 1.21	44.61 ± 4.92	25.52 ± 1.17	17.37 ± 1.22	5.44 ± 0.57	2.31 ± 0.42	3.20
RIL06	25.15 ± 2.83	37.03 ± 2.61	22.59 ± 1.97	16.26 ± 1.53	5.42 ± 0.54	0.91 ± 0.13	3.00
LUOHUI 9	28.06 ± 3.12	27.99 ± 3.33	17.61 ± 0.83	11.03 ± 0.59	4.97 ± 0.46	1.61 ± 0.58	2.22

The cell wall structure of the five straws (offspring) selected fully reflects the difference of parent's character. RIL57 had highest cellulose content (44.61%) with high lignin 22.08%. RIL269 had higher cellulose, lower lignin and hemicellulose (Table 1). Some reports revealed that the degree of branching X/A (xylose/arabinose) of hemicellulose was also one of important factors affecting the enzymatic hydrolysis process (Wu et al., 2013; Fabio et al., 2017). Here, the X/A values of RIL269 and RIL31 were also the lowest. Therefore, we combined these varieties to study the optimal pretreatment conditions for rice straws.

### The Lignin of Different Rice Produces Different Effects in the Pretreatment

In previous studies, acid pretreatment was considered to have a high glucose yield at a concentration of 2% H_2_SO_4_ (Chen et al., 2011), while the alkali concentration needs to be higher (10% NaOH) (Coimbra et al., 2016). Therefore, we designed six sets of different gradient pretreatment methods and concentration comparisons with 1, 2, 4% H_2_SO_4_ and 2, 5, 10% NaOH respectively, for each sample. In addition, the glucose yield of each sample after different pretreatments was further detected by enzymatic hydrolysis.

The glucose yield of each RILs after acid treatment was different. The *japonica* RPY GENG had maximum glucose yield of 70.22% under 2% H_2_SO_4_ treatment, but the glucose yield decreased after the higher acid concentration i.e., 4% H_2_SO_4_ treatment. The glucose yield of the *Indica* LUOHUI 9 was positively correlated with the acid concentration. Although higher concentrations of acid produced higher glucose yields, their efficacy is low with only 43.87% under 1% of H_2_SO_4_. The least change in acid pretreatment concentration was RIL57 with only changes by 12.03% under different H_2_SO_4_ concentrations, while LUOHUI 9 released a maximum glucose increase of 25.36% in the change of acid treatment concentration (Table 2). In addition, the majority of materials with low lignin content were more sensitive to 2% H_2_SO_4_ pretreatment.

**Table 2:** The glucose yield of each group of samples after pretreatment.

**Sample**	**1% H_**2**_SO_**4**_**	**2% H_**2**_SO_**4**_**	**4% H_**2**_SO_**4**_**	**2% NaOH**	**5% NaOH**	**10% NaOH**
RPY GENG	52.93 ± 4.73	70.22 ± 4.38	58.83 ± 1.09	63.93 ± 3.44	69.30 ± 3.18	72.95 ± 5.92
RIL269	48.54 ± 5.29	64.31 ± 5.93	55.06 ± 3.83	60.48 ± 3.90	66.73 ± 4.16	69.06 ± 3.72
RIL272	51.10 ± 3.99	69.03 ± 2.82	60.14 ± 3.27	57.49 ± 5.78	66.62 ± 3.81	71.37 ± 4.63
RIL31	46.50 ± 3.38	58.40 ± 2.22	61.68 ± 3.01	48.09 ± 4.21	61.09 ± 1.30	69.30 ± 3.05
RIL57	42.54 ± 2.48	46.99 ± 3.21	54.57 ± 5.15	47.61 ± 3.26	57.33 ± 3.71	66.66 ± 3.66
RIL06	43.47 ± 3.47	55.30 ± 4.12	63.47 ± 2.72	50.26 ± 3.68	58.53 ± 3.17	67.66 ± 2.68
LUOHUI 9	43.87 ± 2.58	53.46 ± 3.40	69.23 ± 4.35	49.23 ± 2.58	57.72 ± 2.62	68.93 ± 3.67

Compared to acid treatment, the rice straw samples pretreated with NaOH showed better results, and the glucose yield was higher under low concentration of 2% NaOH. The most impressive glucose acquisition rate was RPY GENG (72.95%) and RIL272 (71.37%) treated with 10% NaOH while both had low lignin content (Table 2). Glucose yield of all samples was positively correlated with the NaOH concentration, but the change trend was limited. The increase in RPY GENG glucose yields from 5% NaOH to 10% NaOH was only 3.65%, and the highest increase of 9.33 and 9.13% was recorded in RIL57 and RIL06, respectively. Interestingly, both RIL57 and RIL06 have higher lignin content. Although the trends of NaOH and H_2_SO_4_ treatment were different, the effects of alkali were significantly better than dilute acid treatment (Table 2). The increase in alkali concentration was positively correlated with glucose yield. In particular, increase in glucose yield of RIL31 from 2% NaOH to 5% NaOH was only an increase 13%, which meant that changes in low concentrations of alkali were easier to obtain an increase in glucose yield. With exaltation in NaOH from 2 to 10%, the highest and lowest glucose yield increment in all samples were RIL31 (21.20%) and RIL269 (8.58%) (Table 2).

**Figure 2:**
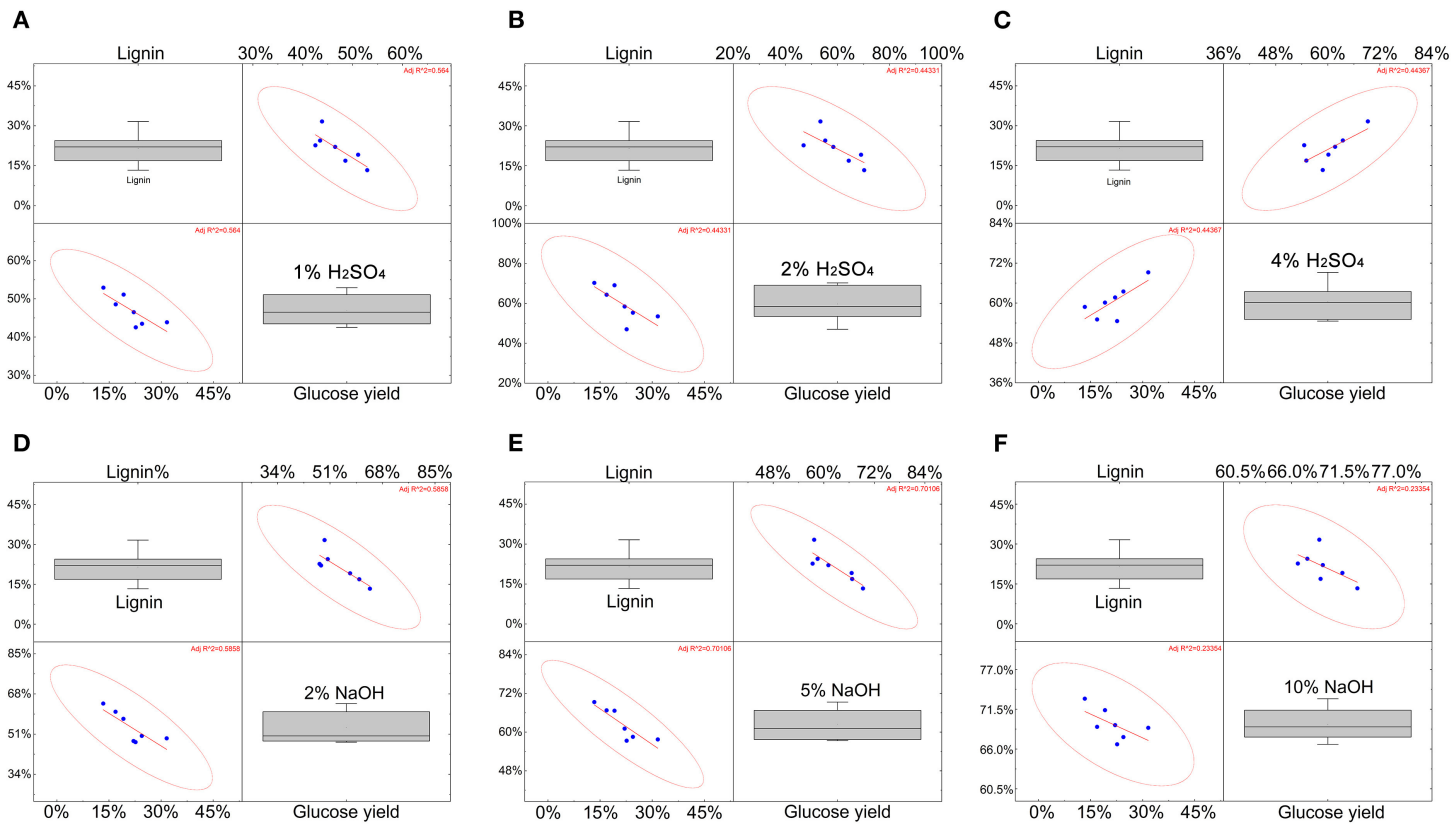
The correlation of the effect of lignin on glucose yield under different concentrations of H_2_SO_4_ and NaOH. **(A–C)** The correlation between lignin and yield under H_2_SO_4_ conditions. **(D–F)** The correlation between lignin and yield under NaOH conditions. The data for each blue point comes from the average of three repeated data. The red line represents the trend, and the red circle represents the degree of fit. The coordinate axes respectively indicate the lignin content and glucose yield.

In previous studies, the lignin content on the enzymatic hydrolysis process was considered to be one of the most key factors (Xu and Henkemeyer, 2012; Li et al., 2014; Sun et al., 2015; Jiang et al., 2016; Wu et al., 2019), that remained to be discussed and the correlation of lignin at different concentrations of H_2_SO_4_ and NaOH were analyzed. Lignin content with 1% H_2_SO_4_, 2% H_2_SO_4_ show a moderate negative correlation, which is consistent with the conclusion in the literature that lignin will affect the pretreatment effect (Li et al., 2010). As the acid treatment concentration increased (4%), the relationship between lignin content and sugar yield turned into a moderately positive correlation. The increase in alkali treatment concentration shows that lignin was negatively correlated with sugar production, but when the NaOH was up to 10%, the correlation was not statistically significant (R squared <0.3). This means that the content of lignin may become less critical under higher-concentration alkali treatment conditions (Figure 2).

**Figure 3:**
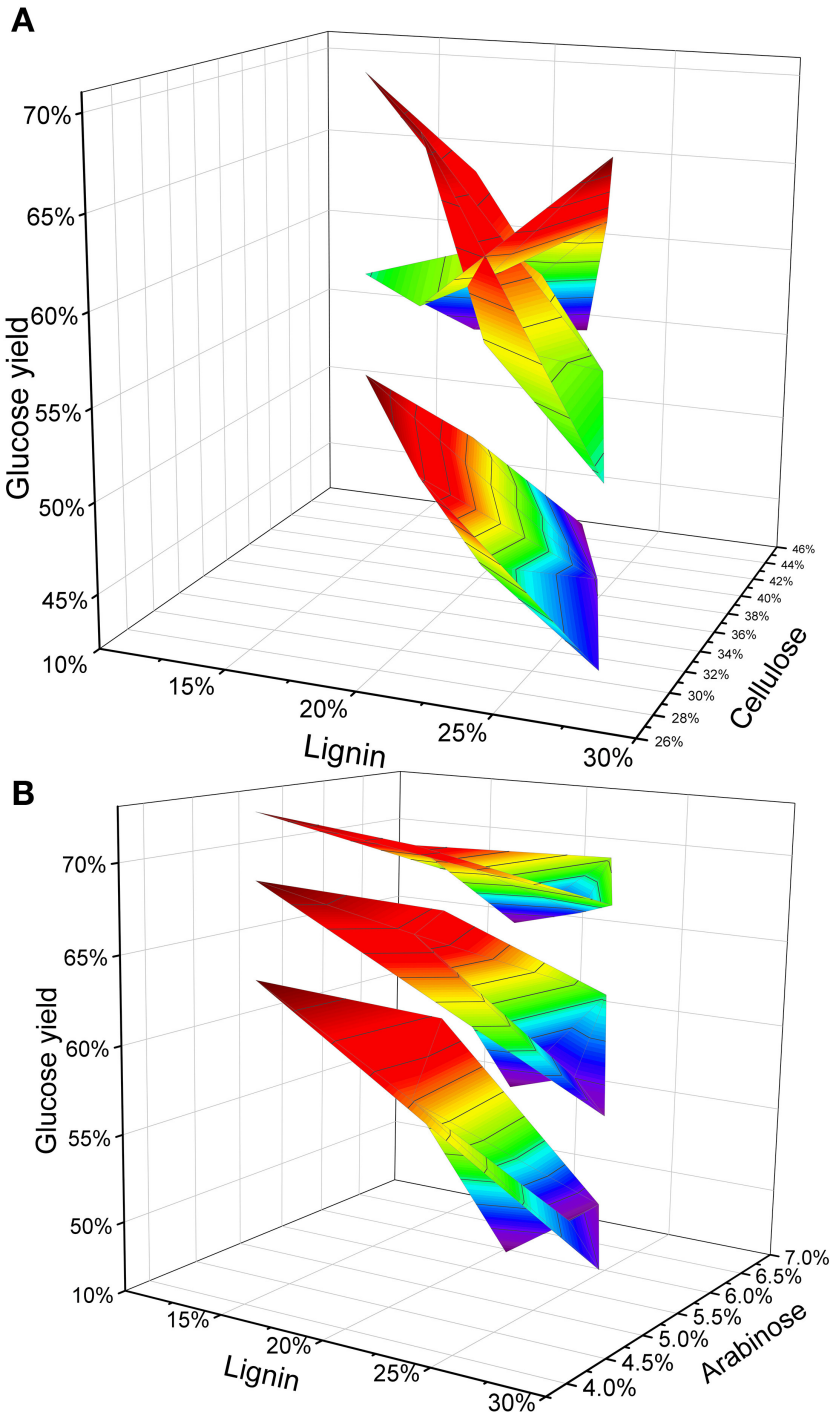
The response trend of lignin combined with other cell wall components to pretreatment. **(A)** Under different acid concentration pretreatments, lignin and cellulose are used as independent variables to obtain the trend of glucose yield. **(B)** Under the pretreatment with different alkali concentrations, lignin and arabinose were used as independent variables to obtain the trend of glucose yield.

Furthermore, we combined three factors that have been reported to affect enzymatic hydrolysis, and explored the overall trend of the five samples under pretreatment conditions. Previously reported results indicate that the structure of cellulose and hemicellulose has an impact on the results of enzymatic saccharification (Lenting and Warmoeskerken, 2001; Qian et al., 2005; Fabio et al., 2017). The H_2_SO_4_ trend response graph shows that straw with different cell wall composition responds differently to H_2_SO_4_. After treatment with 1% H_2_SO_4_, the increase in the amount of reducing sugar obtained from straw was lower than that with 2 and 4% acid treatment. But when the straw has low lignin and high cellulose, the effect of 1% H_2_SO_4_ treatment was equivalent to the 2% H_2_SO_4_ treatment. A low concentration (1%) of H_2_SO_4_, the glucose yields as well as cellulose content gradually decreased and lignin content increased (Figure 3A). Therefore, the sample RIL57 with high lignin and cellulose content did not achieve greater glucose yield at low acid concentrations. In alkali treatment, the lignin content was negatively correlated with glucose yield. Increasing the concentration of alkali is an effective way to increase the yield of glucose. However, the difference in lignin content leads to differential degree of increase in glucose yield. Increased glucose yield of low-lignin straw under different NaOH treatment was much lower than that of high-lignin straw samples and, with further increase in alkali concentrations, this increase has become smaller and smaller (Figure 3B). In particular, When the alkali concentration reaches 10%, the correlation becomes weaker and weaker. As mentioned above, alkaline pretreatment and lignin show a negative correlation. However, when the type of straw is not clear (such as LUOHUI 9 with high lignin and RPY GENG with low lignin), using a high concentration of alkali to treat mixed straw seems to be a more convenient method.

## Discussion

Biologically complex organisms have led to the diversity of biomass energy. As a huge biomass resource, rice straw has been exploited through biotechnology, optimized technology and other methods. *Indica*-*Japonica* hybrid rice recombinant inbred lines are undoubtedly an excellent natural population for studying the relationship between cell wall structure differences and pretreatment effects. The seven samples, with obvious differences in cell wall structure were selected in the present study and have different performances on different pre-treatments, which means that the same pretreatment may not be appropriate for all types of straw.

Previous studies have shown that the stubbornness of the cell wall structure from different varieties is different (Alam et al., 2019). Therefore, there is the possibility of different treatment effects in *indica*-*japonica* hybrid rice. RPY GENG straw has a lower lignin content, and low-concentration acid is more easy to damage the cell wall, which shows that RPY GENG straw has the highest glucose yield under 2% H_2_SO_4_ treatment (Li et al., 2010). Moreover, studies have shown that a small amount of decomposed lignin can effectively inhibit cellulase activity (Kumar et al., 2010; Li et al., 2016). The lower lignin composition reduces the stubbornness of the cell wall while exposing more cellulose that can be hydrolyzed by enzymes. Therefore, a lower concentration of acid treatment of RPY GENG can achieve better results. It is worth noting that chemical pretreatment has unique effects and shortcomings. Therefore, the use of cleaning methods similar to ball milling and biotechnology in RPY GENG is still worth discussing. The glucose yield of LUOHUI 9 with high lignin content after 1% H_2_SO_4_ treatment was only 43.87% (Table 2). So the higher lignin of the samples RIL31, RIL57, RIL06, and LUOHUI 9 makes it more difficult for pretreatment to destroy the cell wall, which affects the yield of sugars (Zhang et al., 2011; Shirkavand et al., 2016). Interestingly, under higher acid treatment conditions, the glucose yield of straw with low lignin content was decreased, while the glucose yields of straw with higher lignin content such as LUOHUI 9 were continued to increase. The reason might be due to the presence of strong acid, the porosity of the cell wall is fully opened, which makes LUOHUI9 have the best acid pretreatment concentration. Meanwhile, the structure of cellulose can be divided into crystalline and amorphous regions, and the amorphous regions are more easily decomposed (Brandt et al., 2013). So, RYP GENG with low lignin content lacks the stubbornness of the cell wall, and seems to be easier to lose cellulose under strong acid concentrations. It has been reported that the crystallinity of sample cellulose after acid treatment has been significantly increased (Sun et al., 2014), making subsequent enzymatic hydrolysis difficult. Not only that, because the concentration of acid pretreatment is too high, the cellulose in the cell wall of straw with low lignin content may be decomposed from the amorphous area, and cause losses. This leads to the loss of the final glucose yield, as in the sample RIL269. In addition, the presence of H bonds makes cellulose more sensitive to acids (Qian et al., 2005). A large amount of lignin in the straw wraps with cellulose to form a strong barrier (Lee et al., 2014; Shirkavand et al., 2016), and high concentration acid is not easy to decompose cellulose, but at the same time, pretreatment destroys the cell wall to a certain extent and increases the accessibility of enzymes, which makes the yield of enzymatic hydrolysis products gradually increase. These factors all indicate that the presence of lignin protects the cell wall from damage under strong acid conditions within a certain range and avoids excessive cellulose loss. Therefore, our selected samples confirmed that rice straw should be suitable for different pretreatment methods according to cell wall composition, such as RPY GENG is suitable for low concentration acid and CHAO2R is more suitable for high concentration acid. Similar results were also shown in transgenic rice using biotechnology to modify the cell wall. The cell wall improved the accessibility of enzymes under optimal pretreatment conditions, thereby enhancing the saccharification effect (Li et al., 2018). In the case of different concentrations of acid treatment, RIL57 showed a low incremental glucose yield, which means that RIL57 is tolerant to changes in acid treatment concentration. Therefore, for the sample RIL57, alkali treatment should be considered to improve the glucose yield. Surprisingly, RIL57 has a relatively high content of cellulose, hemicellulose and lignin, as well as a high galactose content and an X/A ratio, but the glucose yield of each group of acid treatments is very low, which means that pretreatment methods suitable for RIL57 are still worth exploring. Besides, the degree of branching of hemicellulose may be one of the factors affecting the looseness of the cell wall structure (Taherzadeh and Karimi, 2007; Li et al., 2015; Fabio et al., 2017). Results for Glu/Xyl in the population were different. The effect of hemicellulose on enzymatic hydrolysis is generally considered to be negatively correlated (Kumar et al., 2009), but there are also reports that the pretreatment process will remove hemicellulose and increase the accessibility of cellulase (Weiss et al., 2010). Therefore, the influence of straws with different cell wall structures on pretreatment and enzymatic hydrolysis is still a huge challenge.

Both lignin and hemicellulose can be partially dissolved in alkali. Alkaline pretreatment effectively removes hemicellulose and lignin, and can swell the surface (Garcia et al., 2013), thereby increasing exposure area for further enzymatic hydrolysis. Compared with alkali, the dissolution of lignin in acid is negligible. Therefore, the swelled straw surface has sufficient space to increase the accessibility of enzymes. Earlier research results showed that the cell wall gap caused by alkali treatment was significantly larger than other pretreatments (Alam et al., 2019). Therefore, for CHAO2R with high lignin content, alkali treatment may be more suitable. This leads to the same level of alkali treatment that is better than acid. Alkali treatment can reduce the crystallinity of cellulose, but the effective alkali treatment concentration seems to have an upper limit. The greater the alkali concentration, the less the effect of lignin in the straw on the glucose yield. Although the combination of Ara (Arabinose) and FA (Ferulic acid) is thought to be one of the connecting means of hemicellulose and lignin, thereby affect the stability of the cell wall structure (Marriott et al., 2016). However, the correlation of arabinose content under alkaline pretreatment is not obvious. The methods and conditions of pretreatment may cause changes in arabinose retention in the cell wall. The influence of X/A on enzymatic hydrolysis had many opinions in previous studies (Taherzadeh and Karimi, 2007; Wu et al., 2013; Fabio et al., 2017). In these 7 RIL straws, the ratio of X/A does not have a significant effect on the final yield, such as RIL57, indicating that X/A may change in other pretreatment methods and affect enzymatic hydrolysis (Sun et al., 2020).

In short, varieties of rice straws might lead to different adaptability of pretreatment methods according to cell wall compositions. For *japonica* rice RPY GENG, 2% H_2_SO_4_ can be used to obtain the best pretreatment effect. Excessive acid concentration will cause damage and loss, while *indica* LUOHUI 9 is only suitable for high acid pretreatment. In addition, RIL57 has a high content of each cell wall component, but this type of straw is not suitable for acid pretreatment. The maximum increase in glucose yield can only be achieved under conditions of high alkali concentration. High concentration alkali treatment may be the best convenient and efficient pretreatment method for the production of straw mixed with unknown species. According to our results, we can understand the impact of different compositions on final glucose yield.

## Methods

### Material Acquisition and Preparation

The source of the plant materials and the group preparation were from the experimental field of the School of Life Sciences of Wuhan University (Wuhan). The first generation of parental *japonica* rice RPY GENG was crossed with *indica* rice Luohui9, the F1 progeny were selfed, and the second to eleventh generations were all single-grained breeding. A total of 270 samples were taken from a single plant at the mature stage. The leaves and sheaths were removed from the stems, and then dried at 60°C for 24 h, then the dried straw pieces were crushed by a crusher for 1 min and sieved with a sieve (40–60 mesh) for further analysis. The sample was dewaxed with 2:1 (v/v) acetone-ethanol at 90°C by Soxhlet extraction for 5 h, and then air-dried at room temperature to obtain an alcohol-insoluble residue (AIR). The acid concentrations used in the treatment are: 1%H_2_SO_4_, 2%H_2_SO_4_, and 4%H_2_SO_4_; the alkali concentrations used in the treatment are: 2%NaOH, 5%NaOH, and 10NaOH%. The processing time is 3 h, and the mixture is continuously mixed on a shaker. The temperature during shaking was set to 60°C.

## Material Composition Analysis

Cell wall composition analysis was performed using a stepwise acid hydrolysis method. 0.3 g of AIR was taken and acidified by using 72% sulfuric acid (3 ml) at 30°C for 1 h, and then treated at 121°C for 1 h with the addition of 84 ml of deionized water. After cooling, it was filtered using a sand core funnel (G4). The diluted filtrate and the fine res idue were filtered through 0.22 μm nylon membrane for monosaccharide composition analysis by ion chromatography. The ion chromatogram (Metrohm 940, Switzerland) equipped with the CarboPac PA10 column (2 × 250 mm, Dionex, US) and coupled with a PAD detector for quantification was used. Twenty millimeter NaOH was added as an isocratic eluent and eluted at a flow rate of 0.5 mL/min for 20 min. The program was set to 0–75 mM NaAc 15 min for gradient elution, 200 mM NaOH 10 min for washing, and then 20 mM NaOH for re-equilibration. A calibration curve was established to calculate the quantification of the monosaccharide.

The acidified elute was diluted 10 times and the percentage of the sample content was calculated by the following formula:

$$\begin{array}{l} \%\text{Content}=\left(\text{D}\times {\text{V}}_{1}\times 10^{-6}\;\times \text{P}\times 0.9\right)/\text{M}\times 100\% \end{array}$$

where D was the dilution factor of acidified elute (10×), V_1_ was total liquid volume used for hydrolysis (V = 86.663 ml), P was the concentration determined by ion chromatography (mg/L) and the M was weight of AIR.

The lignin determination method of 270 samples of *indica* rice and *japonica* rice stalks from the hybrid progeny adopted the NREL standard procedure. Take 0.3 g of AIR and acidify with 72% sulfuric acid (3 ml) at 30°C for 1 h, and then add 84 ml of deionized water for treatment at 121°C for 1 h. After cooling, filter with sand core funnel (G4). G4 sand core needs to be dried to constant weight before use, and its weight is recorded as m_1_. The G4 sand core funnel with filter residue after filtration is also placed in the oven to dry and the constant weight is recorded as m_2_, the weight of acid-insoluble lignin (AIL) is (m_2_-m_1_-weight of ash). The filtrate was then filtered through a 0.22 μm filter membrane, using 4% sulfuric acid solution as a blank control and calibration, and the absorbance of the filtrate was measured on an ultraviolet spectrophotometer. According to the NREL standard, gramineous plants such as corn and rice need to be measured at a wavelength of 320 nm, and the calibration coefficient used is 55. The acid-soluble lignin (ASL) content ratio can be obtained by substituting the value into the following formula:

$$\begin{array}{l} ASL\%=\left(0.08673\times \text{Absorbance}\right)÷\left(55\times M_{AIR}\right)\times \;100\% \end{array}$$

M_AIR_ here refers to the weight of the acid hydrolyzed AIR sample used. The lignin content of the sample is the sum of acid-soluble lignin (ASL) and acid-insoluble lignin (AIL).

### Correction of Ash Content

The determination of lignin corrects the ash content. A muffle furnace was used for ash measurement. Use fast ashing method. Before the measurement, the ash dish was burned to a constant weight, and then 1 g of the AIR sample was evenly distributed in the container. Slowly push the sootware into the muffle furnace at 850°C and burn it for 40 min. Record the weight after cooling to room temperature.

### Enzymatic Hydrolysis

The hydrolyzed samples consisted of AIR and pretreated AIR by 2% NaOH (60°C, 6 h). 0.3 g of AIR was dissolved in 30 ml 0.1 M NaAC-HAC buffer (pH = 4.5). The cellulase (HESHIBI, Complex enzymes such as exo β-glucosidase and endo β-glucosidase, 20 FPU/g) was chosen for the enzymatic hydrolysis analysis at 55°C for 48 h. After the enzymatic hydrolysis, the reaction liquid was deactivated and filtered through a 0.22 μm filter. The concentrations of glucose were determined by ion chromatography. The hydrolysis efficiency of the enzyme was calculated as follows:

$$\begin{array}{l} \%\text{Glucoseyield}=\left(\left(\text{D}\times \text{R}\times {\text{V}}_{2}\right)\right)/\left(\left(\text{P}\times {\text{V}}_{1}\right)\right)\;\times 100\% \end{array}$$

where R was the released glucose concentration by ion chromatography. V_2_ was a volume of the enzymatic reaction liquid. V_1_, D and P have the same meanings as above.

### Analysis of Data

The data were entered into Excel (Microsoft Office® 2019) tables for data processing. Origin (OriginLab, Northampton, Massachusetts, USA) was used for mapping and fitting.

## Data Availability Statement

The original contributions presented in the study are included in the article/Supplementary Materials, further inquiries can be directed to the corresponding author/s.

## Author Contributions

A-MW, YL, CC, MQ, and SZ: designed the study and wrote the manuscript. CC, XD, and WK: performed the experiments and analyzed the data. XD and WK: techniques' help. YL: developed the rice population. All authors contributed to the article and approved the submitted version.

## Conflict of Interest

The authors declare that the research was conducted in the absence of any commercial or financial relationships that could be construed as a potential conflict of interest.

## Supplementary Material

Supplementary Figure 1 The distribution of stalk traits of 270 samples in the population.

Supplementary Figure 2 The correlation between the different traits of the population cell wall is weak.
