# Supplementary figures and images for: Rice Straws With Different Cell Wall Components Differ on Abilities of Saccharification
Source: Front Bioeng Biotechnol. 2021 Jan 20;8:624314. doi: 10.3389/fbioe.2020.624314 (PMC7855461; doi:10.3389/fbioe.2020.624314)

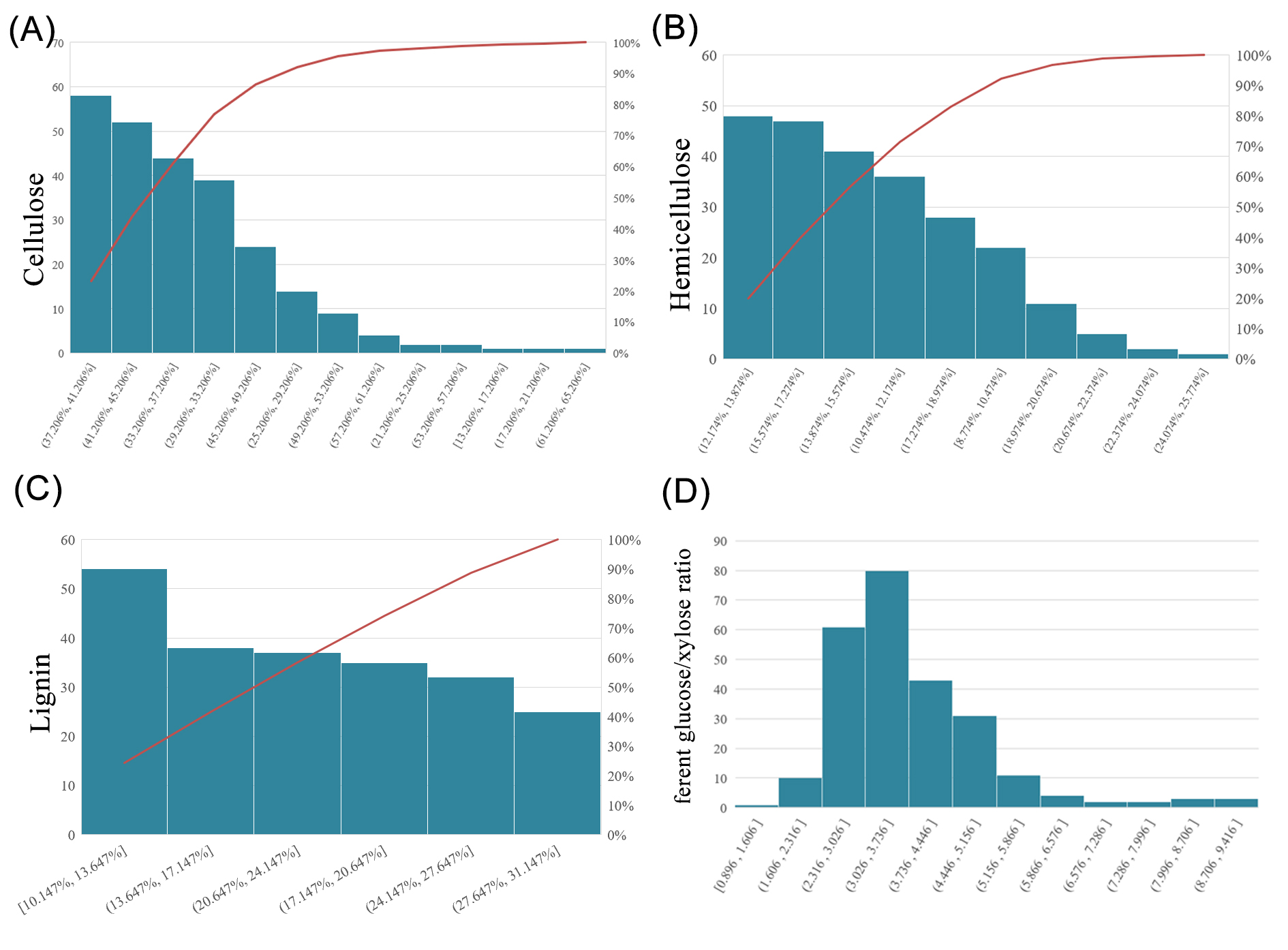

Supplement: Supplementary Figure 1 — The distribution of stalk traits of 270 samples in the population. [file Image_1.JPEG]

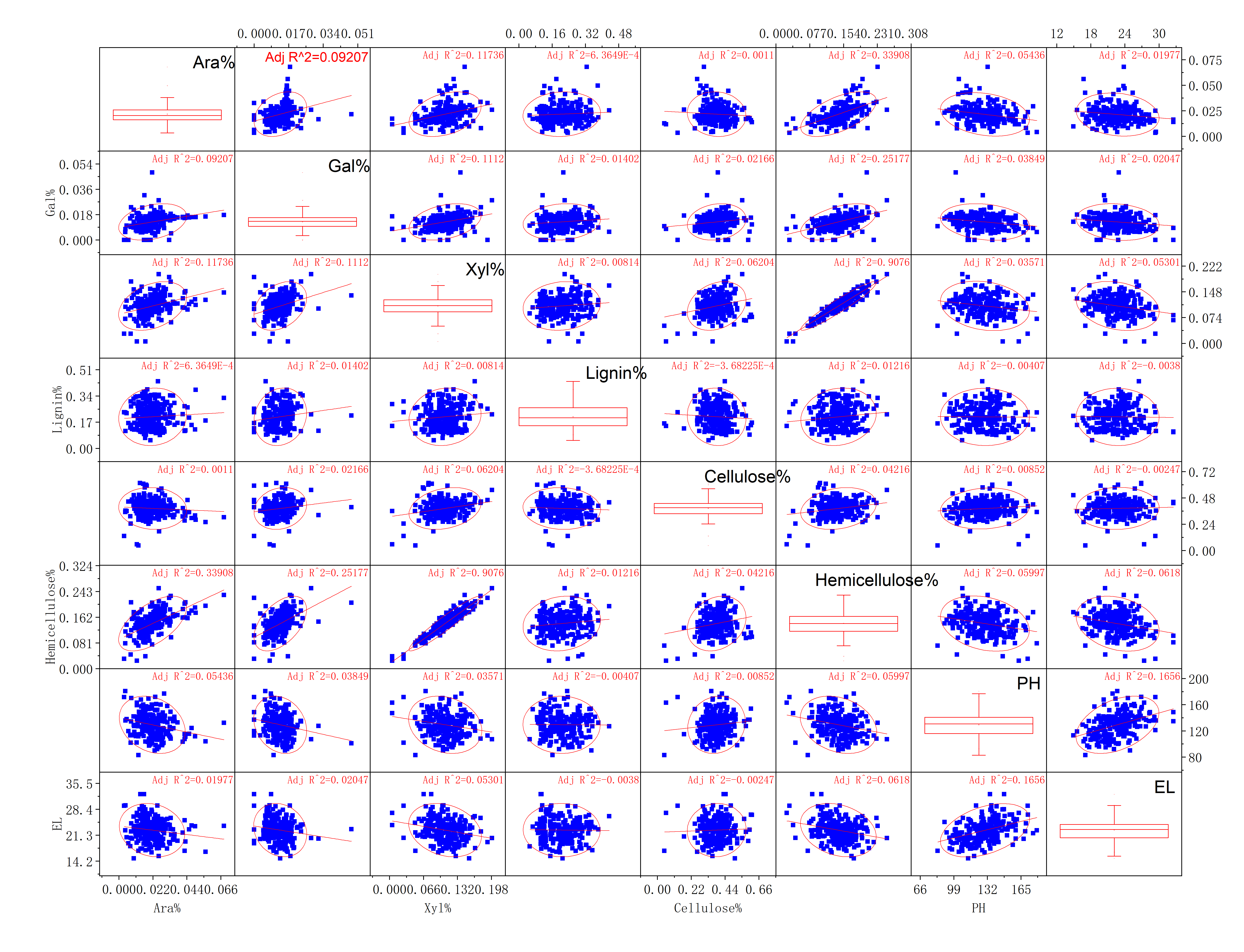

Supplement: Supplementary Figure 2 — The correlation between the different traits of the population cell wall is weak. [file Image_2.PNG]
